# Going with the flow: endothelial dysfunction in type 2 diabetes

**DOI:** 10.1152/function.037.2026

**Published:** 2026-06-05

**Authors:** Luke S. Dunaway

**Affiliations:** Department of Physiology, Tulane University School of Medicine, New Orleans, Louisiana, United States

A healthy cardiovascular system requires a healthy endothelium. The endothelium is a central regulator of hemostasis, vascular permeability, inflammation, blood pressure, and, perhaps most fundamentally, blood flow (1). It is therefore of no surprise that endothelial dysfunction underlies many of the vascular complications that occur in metabolic disease (e.g., peripheral artery disease, vascular retinopathy, and coronary artery disease) (2). Endothelial function is often assessed using flow-mediated dilation (FMD) (3), and in this journal, Power et al. (4) provide novel insight into the mechanisms by which FMD is impaired in type 2 diabetes (T2D).

**Figure 1.**
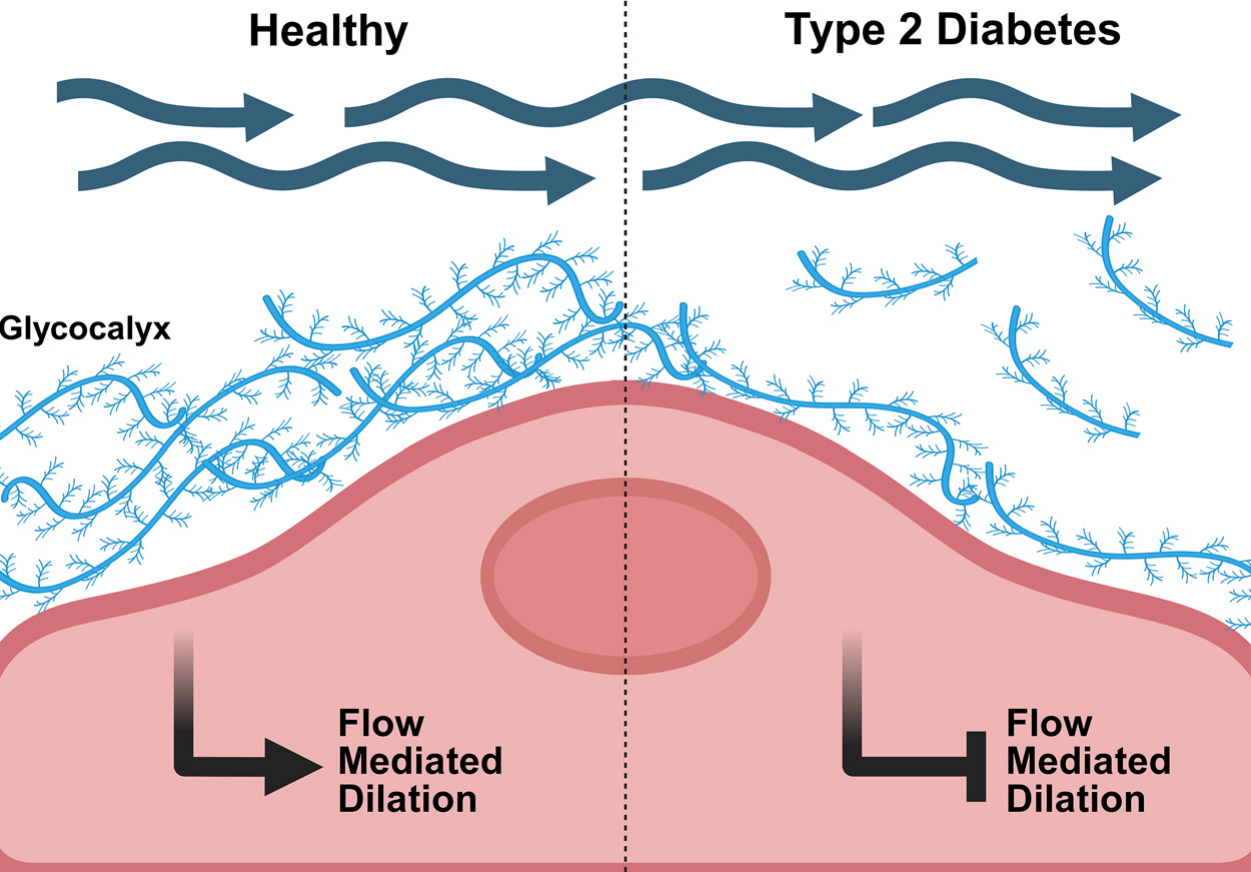
Loss of the glycocalyx blunts flow-mediated dilation in type 2 diabetes. Previous work has established that the endothelial glycocalyx is shed in type 2 diabetes (5). In their recent work, Power et al. (4) provide novel insights into how glycocalyx-dependent mechanotransduction is impaired in type 2 diabetes.

In T2D, loss of FMD is driven, in part, by the shedding of the glycocalyx (Fig. 1). This hair-like structure on the surface of the endothelium maintains an erythrocyte-free zone, preserves redox homeostasis, mitigates inflammation, and senses shear stress (5). The glycocalyx is a complex matrix made up of proteoglycans, glycoproteins, glycolipids, and glycosaminoglycans, and each of these components plays an essential role in its functions (5). Power and colleagues found that patients with T2D had impaired FMD and elevated plasma hyaluronan, suggesting glycocalyx shedding. Hyaluronan is a glycosaminoglycan in the glycocalyx that works in concert with CD44 to propagate flow-induced signaling (6). Notably, they found that endothelial CD44 expression was decreased in the db/db model of T2D.

Using isolated mouse mesenteric arteries, they demonstrated cleavage of hyaluronan with hyaluronidase or blocking the binding site of hyaluronan on CD44-blunted FMD. Similarly, knockdown of CD44 in cultured endothelial cells blunted shear stress-induced increases in intracellular calcium and endothelial nitric oxide synthase. Together, these data demonstrate the importance of hyaluronan-CD44 signaling for endothelial mechanotransduction.

Building on their previous work, which reported upregulation of a disintegrin and metalloprotease 17 (ADAM17) in arteries from patients with T2D, they found elevated plasma ADAM17 activity in their patient population and increased endothelial ADAM17 expression in db/db mice. They therefore investigated whether cleavage of CD44 by ADAM17 contributes to impaired FMD in T2D. They found both activation and overexpression of ADAM17 blunted the response to shear stress in cultured endothelial cells. Furthermore, ADAM17 overexpression cleaved CD44 as demonstrated by reduced cell-surface CD44 and increased CD44 in the supernatant. Similar results were achieved when endothelial cells were treated with recombinant ADAM17, and recombinant ADAM17 was sufficient to impair CD44-hyaluronan binding in vitro. Perhaps most importantly, recombinant ADAM17 was sufficient to blunt FMD in isolated mouse mesenteric arteries. Altogether, these data suggest ADAM17 upregulation contributes to the endothelial dysfunction observed in patients with T2D.

These studies build on previous work, implicating ADAM17 as a driver of endothelial dysfunction in metabolic disease and provide a novel mechanism by which ADAM17 impairs endothelial-dependent vasodilation. Previous work has demonstrated that ADAM17 cleaves glypican-1, a proteoglycan component of the glycocalyx, thereby blunting FMD (7), and ADAM17 cleaves the insulin receptor, blunting insulin-dependent vasodilation (8, 9). Still, questions remain regarding how ADAM17 activity impacts other functions of the glycocalyx and how other flow-sensing mechanisms, such as piezo channels, may compensate in these conditions. The use of endothelial-specific ADAM17 knockout mice offers an exciting avenue to pursue these questions in vivo (7).

Altogether, ADAM17 may be a promising target to preserve or restore the endothelial glycocalyx and endothelial function in T2D. Vascular dysfunction gives rise to major T2D comorbidities such as nephropathy, neuropathy, and cardiovascular disease (10). Continued efforts to investigate the mechanisms that drive endothelial dysfunction may offer new therapeutic targets to treat or prevent these comorbidities, thereby decreasing mortality and improving quality of life for patients with T2D.
